# A laryngeal foreign body resembling cervical emphysema

**DOI:** 10.1002/ccr3.6180

**Published:** 2022-08-03

**Authors:** Hironobu Nishiori, Takayuki Tohma, Hisanori Fujita, Seiichi Yamaguchi

**Affiliations:** ^1^ Division of Cardiovascular Surgery Chiba Emergency Medical Center Chiba Japan; ^2^ Division of Acute Care Surgery Chiba Emergency Medical Center Chiba Japan

**Keywords:** cervical emphysema, chest pain, laryngeal foreign body, plate‐shaped grilled meat

## Abstract

An 81‐year‐old woman was referred to our hospital with a chief complaint of chest discomfort; CT imaging suggested for cervical emphysema. However, direct observation revealed a grilled liver stuck to the larynx. Carefully taking the patient's history, especially diet, is important to diagnose a laryngeal foreign body correctly.

## Graphical abstract

Laryngeal foreign bodies from the grilled liver can cause chest discomfort and may resemble cervical emphysema on CT images. Carefully taking the patient's history, especially diet, is important to diagnose a laryngeal foreign body correctly.

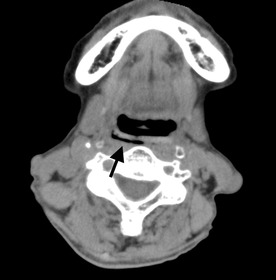

## 1 CASE

**FIGURE 1:**
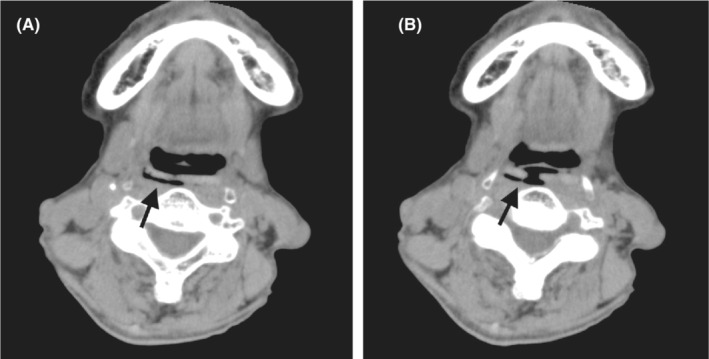
(A) Coronal view of computed tomography imaging showing the presence of air along the posterior wall of the pharynx (arrow). (B) Coronal view of computed tomography imaging showing the presence of air along the posterior wall of the larynx.

**FIGURE 2:**
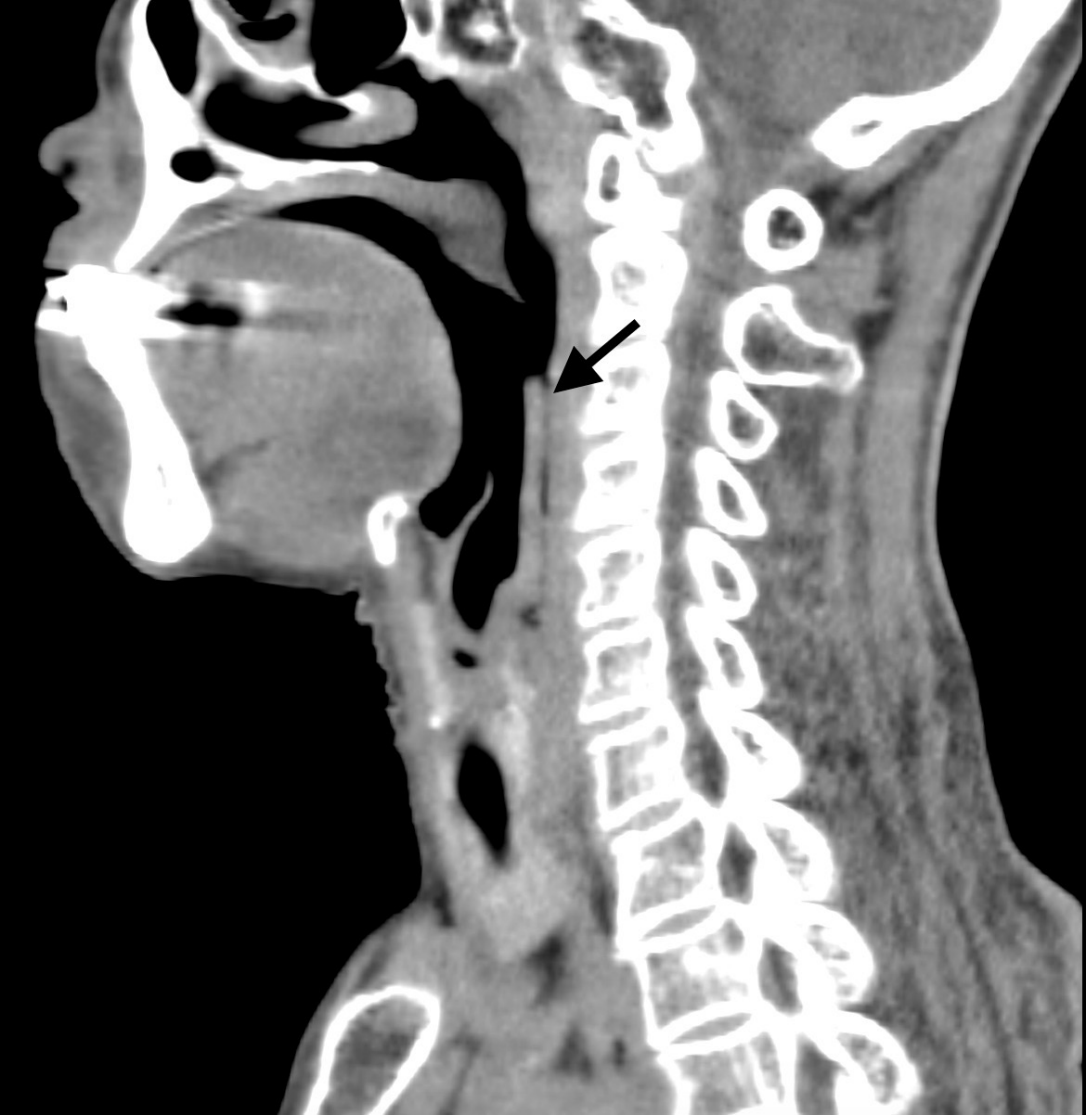
Sagittal view of computed tomography imaging showing the plate‐shaped object adhered to the posterior wall of the pharynx (arrow).

**FIGURE 3:**
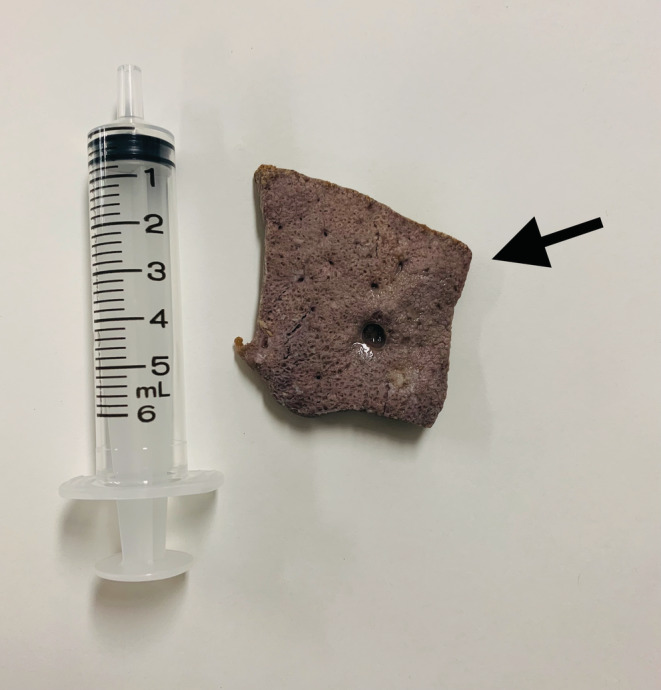
Removed plate‐shaped grilled liver (arrow)

An 81‐year‐old woman with a history of type 2 diabetes mellitus was referred to our hospital, suspecting the cervical emphysema due to esophageal rupture. She developed acute chest discomfort and pain suddenly without signs of respiratory distress during dinner. The cervical computed tomography (CT) images showed the presence of air along the posterior wall of the pharynx, which led the former physician to suspect the cervical emphysema. (Figure 1A,B) We found a sagittal view of CT imaging showed a plate‐shaped object attached to the posterior wall of the larynx (Figure 2). Using a laryngoscope, we found a plate‐shaped grilled liver attached to the larynx wall and removed it with forceps (Figure 3). After the removal, her symptoms resolved without relapse.

**FIGURE 4:**
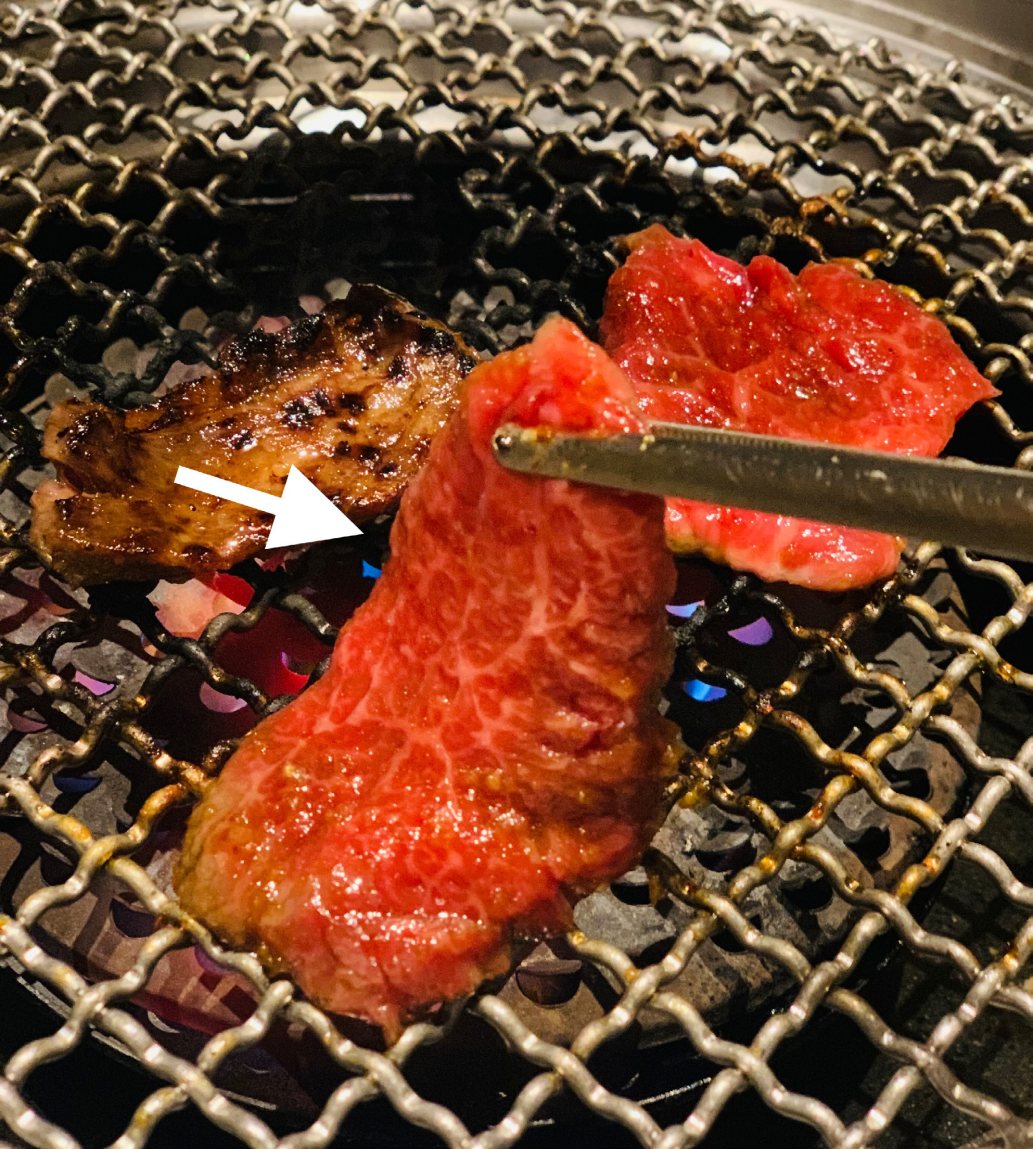
Japanese style plated‐shaped grilled meat called “Yakiniku”

Laryngeal foreign bodies are relatively rare in adults but can occur in the elderly with few molars or poor coordination of swallowing.^1^ The “steak house syndrome” refers to esophageal food impaction, which can be mistaken as esophageal cancer on CT imaging.^2^ In Japan, eating plate‐shaped grilled meat called “Yakiniku” is popular (Figure 4). As in this case, when the grilled liver adheres to the larynx wall, the CT images can resemble cervical emphysema, which occurs secondary to retropharyngeal abscess or esophageal perforation. Carefully taking the patient's history, especially the symptom onset and diet, is important to diagnose a laryngeal foreign body correctly.

## AUTHOR CONTRIBUTIONS

HN and TT cared for the patient. HN got the patient consent form and prepared the clinical picture and computed tomography imaging data, and wrote the report. KT, HF, and SY read and approved the final version of the report.

## CONFLICT OF INTEREST

The authors have no pertinent conflicts of interest to report for this manuscript.

## ETHICAL APPROVAL

None.

## CONSENT

Written informed consent was obtained from the patient to publish this report in accordance with the journal's patient consent policy.

## Data Availability

None

## References

[1] Hada MS , Samdhani S , Chadha V , Harshvardhan RS , Prakash M . Laryngeal foreign bodies among adults. J Bronchology Interv Pulmonol. 2015;22(2):145‐147. 25887012 10.1097/LBR.0000000000000056

[2] Shikino K , Ikusaka M . Steakhouse syndrome. Clin Case Rep. 2021;9(6):e04329. doi:10.1002/ccr3.4329 34136246 PMC8190540

